# Comparative Long-Term Cardiovascular Outcomes of Empagliflozin and Dapagliflozin in Heart Failure Patients After Coronary Revascularization: A Retrospective Cohort Study

**DOI:** 10.3390/jcm14238383

**Published:** 2025-11-26

**Authors:** Ilhan Ozgol, Cennet Yildiz, Ece Yigit Gencer, Dilay Karabulut, Fatma Nihan Turhan Caglar, Burcu Bicakhan, Melek Yilmaz, Umut Karabulut, Yasar Gokkurt, Zerrin Yigit

**Affiliations:** 1 Department of Cardiovascular Surgery, Prof. Dr. Cemil Tascioglu City Hospital, Darülaceze Avenue No. 27, Kaptanpaşa District, 34348 Istanbul, Turkey; opdrmelek@gmail.com (M.Y.); yasargokkurt@hotmail.com (Y.G.); 2 Department of Cardiology, Bakırkoy Dr. Sadi Konuk Training and Research Hospital, 34147 Istanbul, Turkey; cennet_yildiz@live.com (C.Y.); dilay_karakozak@hotmail.com (D.K.); nhnturhan@gmail.com (F.N.T.C.); 3 Department of Internal Medicine, Medipol University Hospital, Faculty of Medicine, Istanbul Medipol University, 34893 Istanbul, Turkey; drece-89@hotmail.com; 4 Department of Cardiovascular Surgery, Gaziosmanpasa Training and Research Hospital, 34255 Istanbul, Turkey; isiksungur@gmail.com; 5 Department of Cardiology, Acıbadem International Hospital, 34149 Istanbul, Turkey; umkarabulut@gmail.com; 6 Department of Cardiology, Faculty of Medicine, Istanbul University Cerrahpasa, 34098 Istanbul, Turkey

**Keywords:** SGLT2 inhibitors, empagliflozin, dapagliflozin, heart failure, coronary revascularization, MACE, atrial fibrillation, Cox regression, real-world study

## Abstract

**Background**: Empagliflozin and dapagliflozin are the most widely prescribed sodium–glucose cotransporter-2 inhibitors (SGLT2i) with established cardioprotective benefits across the spectrum of heart failure (HF). However, direct comparative data remain limited, particularly in patients with a history of coronary revascularization—a population at persistently high cardiovascular (CV) risk. This study aimed to compare the long-term cardiovascular outcomes of empagliflozin versus dapagliflozin in revascularized HF patients who had undergone coronary artery bypass grafting (CABG) or percutaneous coronary intervention (PCI). **Methods**: This retrospective cohort study included 631 HF patients who had undergone coronary revascularization (CABG or PCI) and were treated with an SGLT2 inhibitor (353 dapagliflozin, 278 empagliflozin) between 2014 and 2022 at a tertiary cardiovascular center. Patients were stratified by left ventricular ejection fraction (LVEF ≥ 50%: HFpEF; LVEF < 50%: HFrEF/HFmrEF). The primary outcomes were all-cause mortality, cardiac mortality, major adverse cardiovascular events (MACE), cardiac MACE, and HF-related hospitalization. Cox regression analyses—including time-dependent covariates—were performed to identify independent predictors of cardiac MACE. **Results**: Baseline demographic, clinical, and biochemical characteristics were comparable between groups. During a mean follow-up of 19.6 ± 1.5 months, there were no significant differences between dapagliflozin and empagliflozin in all-cause mortality (19.3% vs. 19.8%), cardiac mortality (11.0% vs. 12.2%), MACE (25.8% vs. 26.3%), cardiac MACE (23.8% vs. 21.9%), or hospitalization (23.8% vs. 23.7%) (all *p* > 0.05). Subgroup analyses by LVEF yielded consistent findings. In time-adjusted Cox modeling, age (HR = 2.089; 95% CI: 1.723–2.533; *p* < 0.001) and atrial fibrillation (AF) (log-rank *p* = 0.030) were identified as significant predictors of cardiac MACE, while creatinine and NT-proBNP lost significance after adjustment. Both age and AF showed time-varying hazard effects, with risk attenuation over time. **Conclusions**: In this real-world cohort of revascularized HF patients, empagliflozin and dapagliflozin demonstrated comparable long-term cardiovascular outcomes, supporting a class effect of SGLT2 inhibitors in this high-risk population. Beyond pharmacologic comparison, age and AF emerged as dynamic predictors of cardiac MACE, highlighting the importance of longitudinal, time-dependent risk assessment in heart failure management following coronary revascularization.

## 1. Introduction

Sodium–glucose cotransporter-2 inhibitors (SGLT2i) selectively and reversibly inhibit SGLT2 in the proximal renal tubules, thereby reducing glucose reabsorption [1,2]. Although initially developed for glycemic control in patients with diabetes, these agents have demonstrated significant cardiovascular (CV) benefits extending beyond glucose regulation in recent years. They markedly reduce the incidence of major adverse cardiovascular events (MACE), cardiovascular mortality, and heart failure (HF)–related hospitalizations [3,4,5,6,7,8,9,10,11,12,13]. Moreover, through a broad range of pleiotropic effects—including natriuresis, modulation of renal hemodynamics, improvement of cardiac energy metabolism, and anti-inflammatory actions—SGLT2 inhibitors exert multi-organ protective roles [14,15].

Among SGLT2 inhibitors, empagliflozin and dapagliflozin are the two most frequently prescribed representatives of the class. Although both agents provide comparable clinical benefits, they exhibit subtle pharmacodynamic differences in SGLT2 selectivity, tissue distribution, and metabolic profile. It has been proposed that these variations might translate into differences in long-term clinical outcomes; however, direct head-to-head comparative evidence remains limited. In particular, real-world data on heart failure patients who have undergone coronary revascularization are extremely scarce.

This retrospective observational cohort study was exploratory and hypothesis-generating in nature. The primary objective was to compare the long-term clinical outcomes of empagliflozin and dapagliflozin in patients with HF who had undergone coronary artery bypass grafting (CABG) or percutaneous coronary intervention (PCI). Specifically, all-cause mortality, cardiac mortality, major adverse cardiovascular events (MACE), and hospitalization rates were evaluated, along with subgroup analyses stratified by left ventricular ejection fraction (LVEF) categories.

## 2. Statistical Analysis

All statistical analyses were conducted using SPSS version 27.0 (IBM Corp., Armonk, NY, USA). Continuous variables were expressed as mean ± standard deviation (SD) or median (interquartile range, IQR), and categorical variables as counts and percentages. For pairwise comparisons, data normality was assessed using the Shapiro–Wilk test and Q–Q plots, while homogeneity of variances was verified with Levene’s test. When both assumptions were satisfied, independent-samples *t*-tests were applied; otherwise, non-parametric tests (e.g., Mann–Whitney U) were used. Categorical variables were compared using the chi-square or Fisher’s exact test, as appropriate.

Prior to survival modeling, the proportional hazards (PH) assumption was evaluated by introducing time-dependent interaction terms for each covariate (Covariate × Survival Time). A significant interaction (*p* < 0.05) was interpreted as a violation of the PH assumption. In this context, atrial fibrillation (AF; interaction *p* = 0.008) and age (interaction *p* < 0.001) exhibited time-varying effects and were addressed using a combined approach: the Cox model was stratified by AF to ensure proportionality within AF strata, while age was retained with its time-dependent covariate. To address this violation, age was modeled using a time-dependent interaction term (TIMEAGE = Age × Survival Time), allowing the hazard associated with age to vary over the follow-up period rather than remain constant. Covariates that satisfied the PH assumption (e.g., creatinine, NT-proBNP) were modeled conventionally.

Univariable Cox regression analyses were first performed to identify potential predictors. Subsequently, multivariable models incorporated clinically relevant covariates (age, AF, creatinine, NT-proBNP) irrespective of their univariable significance, along with the aforementioned time-dependent and stratification components. Effect sizes were reported as hazard ratios (HRs) with corresponding 95% confidence intervals (CIs). Kaplan–Meier curves and the log-rank (Mantel–Cox) test were used to compare survival distributions (e.g., AF vs. non-AF).

Missing data for primary covariates were <5% and considered missing completely at random (MCAR) according to Little’s test; thus, complete-case analyses were performed.

A priori sample size estimation indicated that detecting an absolute difference of ±10% in cardiac MACE between groups with 80% power (two-sided α = 0.05) would require approximately 317 patients per group. The final sample (353 vs. 278 patients) closely approximated this requirement.

All statistical tests were two-tailed, and *p* < 0.05 was considered statistically significant.

## 3. Materials and Methods

### 3.1. Study Design and Population

This retrospective, observational cohort study included a total of 631 patients who met the eligibility criteria, all of whom had a confirmed diagnosis of heart failure (HF) and underwent coronary artery bypass grafting (CABG) or percutaneous coronary intervention (PCI) at a tertiary cardiovascular center between December 2014 and June 2022. All patients were treated with sodium–glucose cotransporter-2 inhibitors (SGLT2i). All patients were initiated on either empagliflozin or dapagliflozin only after the index coronary revascularization procedure, had no prior exposure to SGLT2 inhibitors, and had been receiving the same agent continuously for at least six months at the time of study entry, thereby ensuring a uniform treatment initiation point and minimizing pre-baseline variability. Given that the study period spanned nearly a decade, potential confounding effects arising from changes in treatment guidelines, diagnostic criteria, and prescribing practices over time were carefully considered.

**Figure 1:**
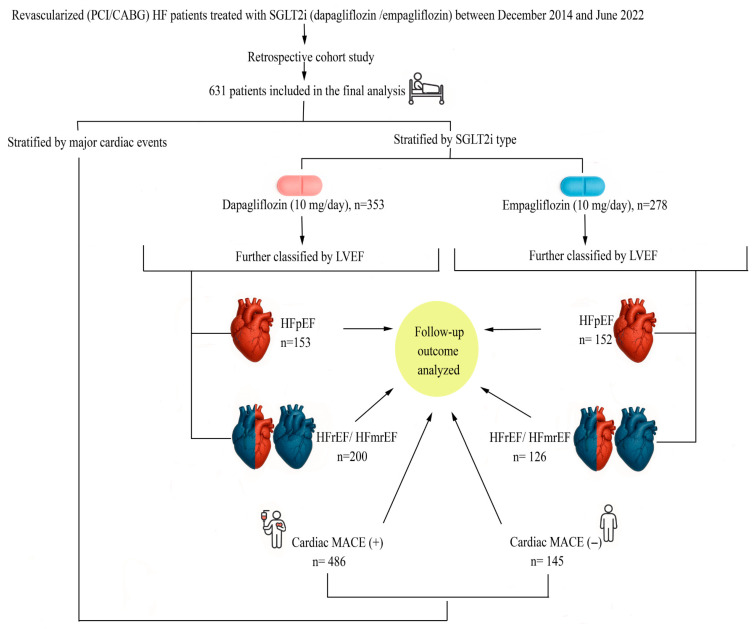
Study flow diagram. Flow diagram illustrating patient inclusion, allocation to SGLT2 inhibitor groups (dapagliflozin vs. empagliflozin), classification according to left ventricular ejection fraction (HFpEF vs. HFrEF/HFmrEF), and stratification by cardiac major adverse cardiovascular events (MACE). Follow-up outcomes were analyzed across all resulting subgroups. Abbreviations: CABG, coronary artery bypass grafting; PCI, percutaneous coronary intervention; HF, heart failure; HFpEF, heart failure with preserved ejection fraction; HFmrEF, heart failure with mildly reduced ejection fraction; HFrEF, heart failure with reduced ejection fraction; LVEF, left ventricular ejection fraction; MACE, major adverse cardiovascular events; SGLT2i, sodium–glucose cotransporter-2 inhibitor.

Patients were eligible for inclusion if they had a confirmed diagnosis of HF in accordance with the European Society of Cardiology (ESC) guidelines, a documented history of CABG and/or PCI, and had been receiving continuous treatment with either empagliflozin (10 mg/day) or dapagliflozin (10 mg/day) for at least six months. Left ventricular ejection fraction (LVEF) was verified by transthoracic echocardiography and classified as either preserved or reduced. Patients were excluded if they were younger than 18 years of age, had type 1 diabetes mellitus, uncontrolled hypertension or hyperglycemia, or a history of alcohol, substance, or corticosteroid abuse. Additionally, patients with moderate-to-severe aortic stenosis, significant hypertensive heart disease, primary cardiomyopathies (dilated, hypertrophic, or restrictive), or a prior history of valvular surgery were excluded due to their potential prognostic impact (Figure 1).

### 3.2. Data Collection and Echocardiographic Assessment

Demographic, clinical, and biochemical data were retrieved from electronic medical records. Information on guideline-directed medical therapy (angiotensin receptor–neprilysin inhibitors (ARNi), beta-blockers, mineralocorticoid receptor antagonists (MRAs), and diuretics) was recorded. All patients received standard therapy according to contemporary guideline recommendations unless contraindicated. Due to the absence of socioeconomic data, details regarding concomitant medications were considered a limitation.

Medication adherence was assessed based on patient self-report. During follow-up, each participant was verbally asked whether they were regularly taking their prescribed medications.

The completeness of primary covariates was evaluated. No missing data were observed for age, whereas fasting glucose values were missing for six patients (0.9%). Since this rate was below the generally accepted 5% threshold, the missing data were assumed to be (MCAR). To confirm this assumption, Little’s MCAR test was applied (χ^2^ = 3.4334, *p* = 0.0639), indicating no statistical evidence of systematic bias in missingness.

All echocardiographic assessments were performed by experienced cardiologists using the biplane Simpson method at end-expiration and in the left lateral decubitus position, in accordance with the American Society of Echocardiography guidelines, using a Vivid E9 echocardiography system (GE Healthcare, Chicago, IL, USA). Interobserver agreement was evaluated using a two-way mixed-effects model with absolute agreement. The analysis demonstrated good consistency, with an intraclass correlation coefficient (ICC) of 0.807 for average measures (95% CI: 0.457–0.932, *p* = 0.002), indicating reliable agreement between observers.

Patients were categorized according to LVEF as follows:

- HFpEF: LVEF ≥ 50%
- HFrEF/HFmrEF: LVEF < 50%

In the HFpEF group, 152 patients received empagliflozin and 153 dapagliflozin; in the HFrEF/HFmrEF group, 126 patients received empagliflozin and 200 dapagliflozin.

#### Laboratory Evaluations and Definitions

Blood samples were collected after 10–12 h of fasting. HbA1c, creatinine, blood urea nitrogen (BUN), uric acid, and NT-proBNP were measured as key biochemical parameters. NT-proBNP levels were analyzed using the electrochemiluminescence immunoassay (ECLIA) method on the Roche Elecsys platform (Roche Diagnostics, Basel, Switzerland). Since all participants were in stable outpatient condition, NT-proBNP measurements were obtained in the ambulatory setting.

Blood pressure was measured twice at 10 min intervals, and the mean value was recorded; the rest period between measurements was applied to allow hemodynamic stabilization and minimize short-term variability in patients with heart failure. Hypertension was defined as blood pressure ≥ 140/90 mmHg, and diabetes mellitus as fasting plasma glucose ≥ 126 mg/dL or postprandial glucose ≥ 200 mg/dL. The estimated glomerular filtration rate (eGFR) was calculated using the Chronic Kidney Disease Epidemiology Collaboration (CKD-EPI) formula. Atrial fibrillation (AF) was diagnosed according to the 2020 ESC criteria.

### 3.3. Outcome Definitions and Subgroup Classification

Major adverse cardiovascular events (MACE) were defined as all-cause mortality, nonfatal myocardial infarction (MI), stroke, or hospitalization due to HF. Cardiac MACE comprised cardiac death, nonfatal MI, and HF-related hospitalization. Cardiac mortality included deaths attributed to HF, MI, or sudden cardiac death.

Patients were further classified according to the occurrence of cardiac MACE: those who developed cardiac MACE (*n* = 145) and those who did not (*n* = 486). These subgroups were used for receiver operating characteristic (ROC) analysis, Kaplan–Meier survival analysis, and Cox regression modeling.

### 3.4. Ethical Approval

The study protocol was approved by the Ethics Committee of Bakırköy Dr. Sadi Konuk Training and Research Hospital (Protocol Code: 2025/02, Decision Number: 2025-01-06, Date of Approval: 8 January 2024). All procedures were conducted in accordance with the principles of the Declaration of Helsinki. Patient confidentiality was maintained by anonymizing all personal data.

## 4. Results

A total of 631 patients with heart failure (HF) and a history of coronary revascularization were included in the analysis. Among them, 353 received dapagliflozin and 278 received empagliflozin therapy. Baseline demographic, clinical, and biochemical characteristics were comparable between the two groups (Table 1). The mean age was 62.1 ± 11.3 years in the dapagliflozin group and 61.3 ± 9.7 years in the empagliflozin group (*p* = 0.388). The sex distribution was similar (61.2% vs. 54.3%; *p* = 0.082). There were no significant differences between the groups in the prevalence of hypertension, diabetes mellitus, atrial fibrillation (AF), smoking, PCI, or CABG (all *p* > 0.05). Echocardiographic and laboratory parameters—including LVEF, HbA1c, lipid profile, BUN, creatinine, uric acid, and NT-proBNP levels—were also comparable.

No missing data were observed for age, while fasting glucose values were missing in six patients (0.9%). As the missing rate was below 5%, the data were assumed to be missing completely at random (MCAR). Little’s MCAR test confirmed this assumption (χ^2^ = 3.4334, *p* = 0.0639).

The use of guideline-directed HF therapies (ACEI/ARB, ARNI, beta-blockers, MRAs, diuretics, and calcium channel blockers) was similar between groups. The prevalence of implantable cardiac devices, including ICD/CRT systems and pacemakers, was also comparable between the dapagliflozin and empagliflozin groups (both *p* > 0.05). The mean follow-up duration did not differ significantly (19.67 ± 1.50 vs. 19.47 ± 1.48 months; *p* = 0.08). During follow-up, rates of hospitalization (23.8% vs. 23.7%), all-cause mortality (19.3% vs. 19.8%), cardiac mortality (11.0% vs. 12.2%), MACE (25.8% vs. 26.3%), and cardiac MACE (23.8% vs. 21.9%) were comparable between the two groups (all *p* > 0.05). Although no significant intergroup differences were observed, the effect sizes were as follows: cardiac MACE (OR = 1.048; 95% CI: 0.700–1.568), hospitalization (OR = 0.839; 95% CI: 0.560–1.256), all-cause mortality (OR = 0.969; 95% CI: 0.626–1.500), cardiac mortality (OR = 1.214; 95% CI: 0.724–2.036), and MACE (OR = 0.863; 95% CI: 0.594–1.275).

In subgroup analyses based on ejection fraction, 305 patients had preserved ejection fraction (HFpEF). Of these, 153 received dapagliflozin and 152 received empagliflozin. The mean ages were 60.9 ± 10.6 and 61.9 ± 9.6 years, respectively (*p* = 0.116), with similar sex distribution (58.2% vs. 49.3%; *p* = 0.122). The prevalence of hypertension, diabetes mellitus, AF, smoking, PCI, and CABG were comparable (all *p* > 0.05). Echocardiographic and laboratory parameters—including LVEF, HbA1c, lipid profile, BUN, creatinine, uric acid, and NT-proBNP levels—did not differ significantly. The mean follow-up duration was similar (19.52 ± 1.43 vs. 19.40 ± 1.34 months; *p* = 0.582). Clinical outcomes during follow-up—including hospitalization (28.1% vs. 19.7%), all-cause mortality (23.5% vs. 15.8%), cardiac mortality (12.4% vs. 7.9%), MACE (28.1% vs. 21.7%), and cardiac MACE (26.8% vs. 17.8%)—were also not significantly different between groups (all *p* > 0.05). The prevalence of implantable cardiac devices (ICD/CRT or pacemaker) was similarly low in both groups, with no significant difference observed (Table 2).

Among patients with reduced or mildly reduced ejection fraction (HFrEF/HFmrEF; *n* = 326), 200 received dapagliflozin and 126 received empagliflozin (Table 3). The mean ages were 63.0 ± 11.8 and 62.8 ± 9.7 years, respectively (*p* = 0.997), and the sex distribution was similar (63.5% vs. 60.3%; *p* = 0.564). Clinical characteristics, laboratory parameters, and NT-proBNP levels (2340 ± 3381 vs. 3045 ± 4433 pg/mL; *p* = 0.728) did not differ significantly. The prevalence of implantable cardiac devices, including ICD/CRT systems (14.0% vs. 17.5%) and pacemaker therapy (11.0% vs. 13.5%), was also comparable (all *p* > 0.05). The mean follow-up duration (19.79 ± 1.55 vs. 19.56 ± 1.63 months; *p* = 0.146) and clinical outcomes—including hospitalization (20.5% vs. 28.6%), all-cause mortality (16.0% vs. 24.6%), cardiac mortality (10.0% vs. 17.5%), MACE (24.0% vs. 31.7%), and cardiac MACE (21.5% vs. 26.9%)—were comparable (all *p* > 0.05).

During follow-up, 145 patients (23%) experienced at least one cardiac MACE, while 486 patients (77%) remained event-free (Table 4). Patients with MACE were older (64.86 ± 10.98 vs. 61.38 ± 10.37 years; *p* = 0.004) and had a higher prevalence of AF (30.3% vs. 20.2%; *p* = 0.010). They also had lower LVEF (44.83 ± 14.63 vs. 47.86 ± 10.21%; *p* = 0.037), higher BUN (22.76 ± 14.01 vs. 19.36 ± 10.96 mg/dL; *p* = 0.001) and creatinine levels (1.24 ± 1.09 vs. 0.99 ± 0.41 mg/dL; *p* = 0.006), and significantly higher NT-proBNP concentrations (3266 ± 4443 vs. 1877 ± 3394 pg/mL; *p* = 0.001). The use of oral anticoagulants was also more frequent in the MACE group (35.9% vs. 21.6%; *p* = 0.001). The prevalence of implantable cardiac devices, including ICD/CRT systems (10.4% vs. 7.5%) and pacemakers (9.8% vs. 8.1%), did not differ significantly between patients with and without cardiac MACE (all *p* > 0.05). There was no difference in event occurrence between dapagliflozin and empagliflozin users (*p* = 0.583).

In univariable Cox regression analysis (Table 5), age (HR = 1.026; 95% CI: 1.011–1.042; *p* = 0.001), AF (HR = 1.452; 95% CI: 1.018–2.207; *p* = 0.040), oral anticoagulant use (HR = 2.029; 95% CI: 1.357–3.034; *p* = 0.001), NT-proBNP (HR = 1.001; 95% CI: 1.001–1.002; *p* = 0.007), and creatinine (HR = 1.141; 95% CI: 1.005–1.294; *p* = 0.041) were identified as significant predictors. LVEF was not significant (*p* = 0.068), and the type of SGLT2 inhibitor (empagliflozin vs. dapagliflozin) was also not associated with outcome (HR = 1.007; 95% CI: 0.725–1.398; *p* = 0.968).

The proportional hazards (PH) assumption test revealed significant violations for age (*p* < 0.001) and AF (*p* = 0.008). Negative beta coefficients (TIMEAGE: B = −0.039; TIMEAF: B = −0.056) indicated that the effect of these variables on event risk diminished over time. The PH assumption was met for creatinine (*p* = 0.389) and NT-proBNP (*p* = 0.259). To account for these time-dependent effects, the model was stratified by AF status, and a time-interaction covariate for age (TIMEAGE = Age × Survival Time) was introduced (Table 6). In this adjusted multivariable model, age remained significant (B = 0.737; HR = 2.089; 95% CI: 1.723–2.533; *p* < 0.001), as did the TIMEAGE term (B = −0.038; HR = 0.963; 95% CI: 0.953–0.973; *p* < 0.001). Creatinine (*p* = 0.256) and NT-proBNP (*p* = 0.444) were not independent predictors.

**Figure 2:**
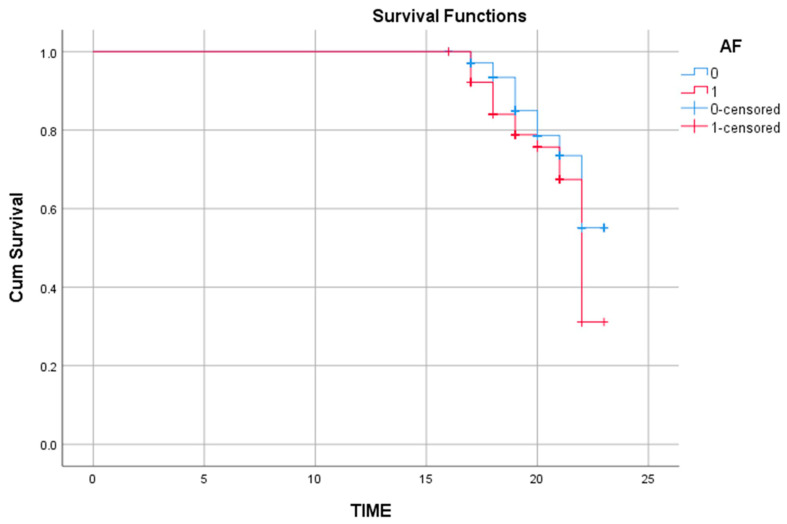
Kaplan–Meier survival curves for cardiac MACE according to atrial fibrillation (AF) status. Kaplan–Meier curves comparing event-free survival between patients with AF (red line) and without AF (blue line). The difference between groups was statistically significant based on the log-rank test. Abbreviations: AF, atrial fibrillation; MACE, major adverse cardiovascular events.

Kaplan–Meier survival analysis was performed to compare survival distributions between patients with and without AF. The log-rank (Mantel–Cox) test demonstrated a statistically significant difference between the two groups (χ^2^(1) = 4.726; *p* = 0.030). The mean survival time was 21.83 units (95% CI: 21.62–22.03) in patients without AF and 21.29 units (95% CI: 20.92–21.67) in those with AF. The median survival time for the AF group was 22.00 units (95% CI: 21.48–22.52). The cumulative hazard curve indicated a higher risk accumulation among AF-positive patients (Figure 2).

## 5. Discussion

Although sodium–glucose cotransporter-2 (SGLT2) inhibitors were originally developed for glycemic control, they have now emerged as a cornerstone therapy in heart failure (HF), demonstrating efficacy regardless of left ventricular ejection fraction (LVEF) status [13]. Landmark randomized controlled trials—including EMPA-REG OUTCOME, EMPEROR-Reduced, EMPEROR-Preserved, DECLARE-TIMI 58, DAPA-HF, and DELIVER—as well as multiple large-scale meta-analyses and real-world observational cohorts, have consistently shown that dapagliflozin and empagliflozin significantly reduce HF-related hospitalizations, cardiovascular (CV) mortality, and all-cause death [4,8,9,10,11,12,13,16,17,18,19]. These robust findings have transformed HF management paradigms, establishing SGLT2 inhibitors as an integral component of current guideline-directed medical therapy.

Beyond their established “class effect,” increasing interest has focused on potential pharmacodynamic distinctions between dapagliflozin and empagliflozin—two of the most widely prescribed agents in this class. Despite similar mechanisms, subtle differences in SGLT2 selectivity, tissue distribution, and metabolic effects may theoretically translate into divergent long-term outcomes. Comparative analyses exploring these aspects, however, have yielded mixed results. Some studies have suggested that dapagliflozin may provide greater reductions in CV mortality, MACE, and HF-related hospitalization compared with empagliflozin [20,21], whereas others have indicated potential advantages for empagliflozin in specific CV outcomes [6,22,23,24,25,26]. Conversely, several head-to-head and network meta-analyses have reported no clinically meaningful differences, supporting the notion of a uniform cardioprotective effect across SGLT2 inhibitors [27,28,29,30,31].

Given these conflicting findings and the limited availability of direct comparative data, our study sought to address this gap using real-world evidence from a revascularized HF cohort. In patients with a history of CABG or PCI, both with preserved and reduced ejection fraction, we found no significant difference between empagliflozin and dapagliflozin in terms of all-cause mortality, cardiac mortality, MACE, cardiac MACE, or hospitalization rates. While no significant difference was observed between the two SGLT2 inhibitors, the study was not powered to exclude smaller effect sizes; therefore, the findings should be interpreted as demonstrating the absence of detectable differences rather than definitive therapeutic equivalence. These results suggest that, in patients with established coronary artery disease and prior revascularization, the cardioprotective benefits of SGLT2 inhibitors can be largely attributed to a class effect, rather than drug-specific properties. Importantly, this study contributes novel comparative insights to a high-risk patient population for whom head-to-head clinical data remain scarce.

LDL-cholesterol levels were relatively high in our cohort despite all patients having established ischemic heart disease. This likely reflects suboptimal statin adherence, variations in statin intensity, and the well-recognized prevalence of residual dyslipidemia in real-world HF populations. As lipid values in this retrospective dataset were obtained during routine outpatient visits rather than standardized follow-up, incomplete lipid optimization may also have contributed

Beyond interdrug comparisons, adverse cardiovascular outcomes in our cohort were more closely associated with advanced age, the presence of atrial fibrillation (AF), and reduced ejection fraction—rather than the type of SGLT2 inhibitor used. This finding emphasizes that patient-level clinical characteristics and comorbidity burden play a more decisive role in long-term prognosis than pharmacologic differences between agents.

In Cox regression analysis, both age and AF emerged as significant predictors of cardiac MACE. Violations of the proportional hazards (PH) assumption for these covariates indicated that their effects on event risk varied over time. Accordingly, age was modeled as a time-dependent covariate (TIMEAGE = Age × Survival Time), while AF was incorporated via a stratified Cox regression to preserve proportionality. In this adjusted model, age remained a strong independent predictor (B = 0.737; HR = 2.089; *p* < 0.001). The negative coefficient of the TIMEAGE term (B = −0.038; HR = 0.963; *p* < 0.001) suggested a diminishing influence of age on event risk over time—potentially reflecting stabilization of initially high-risk older patients during follow-up. Similarly, AF significantly increased event risk (log-rank *p* = 0.030), with Kaplan–Meier analysis demonstrating reduced survival probabilities among AF-positive patients. These results underscore the prognostic impact of AF on long-term outcomes, independent of SGLT2 inhibitor type.

It should also be noted that the higher baseline hazard ratio for age observed in the multivariable model reflects its time-zero effect within the time-dependent framework, while the TIMEAGE interaction term captures the gradual attenuation of this effect as follow-up progresses. Therefore, the age-related risk should be interpreted dynamically rather than as a fixed constant over time.

Conversely, creatinine and NT-proBNP lost significance in the multivariable model (*p* = 0.256 and *p* = 0.444, respectively), likely reflecting their collinearity with other strong predictors such as age and AF. Although elevated in event-positive patients, these biomarkers appear to contribute less prognostic weight once major clinical covariates are accounted for.

Overall, this study not only compares two SGLT2 inhibitors but also introduces a methodological refinement by incorporating time-varying covariates into survival modeling. Accounting for such dynamic effects provides a more realistic representation of long-term cardiovascular risk in real-world settings and may improve predictive accuracy beyond conventional Cox models.

## 6. Limitations and Future Directions

This study has several limitations that warrant consideration. First, its retrospective design inherently limits causal inference and may introduce selection bias. Second, the non-randomized assignment of SGLT2 inhibitors could reflect physician or patient preferences, leading to potential unmeasured confounding. Third, although the sample size was adequate for the primary analyses, it may have been underpowered to detect small intergroup differences between dapagliflozin and empagliflozin. Although the total cohort (*n* = 631) was sufficient to detect large absolute differences in cardiac MACE (±10%), it was underpowered to detect smaller differences (e.g., ±5%), limiting the ability to conclude true equivalence between the two agents. Fourth, while the mean follow-up was approximately 20 months, minor variability (18–23 months) may have introduced residual bias despite censoring adjustments. Although treatment initiation was standardized to the period following coronary revascularization, the exact calendar dates of SGLT2 inhibitor initiation were not uniformly documented, limiting our ability to model treatment duration as a precise time-dependent variable. Although major structural heart diseases were excluded, subtle or subclinical myocardial disorders may not have been fully identifiable in a retrospective design.

Additionally, the definition of MACE in this study included all-cause mortality to provide a comprehensive view of cardiovascular risk, which may limit comparability with studies using narrower or more conventional endpoints. Data on the duration of diabetes—an important determinant of cardiovascular prognosis—were unavailable, potentially affecting the accuracy of risk stratification. Longitudinal renal function parameters were not consistently recorded across follow-up visits, preventing a comprehensive evaluation of renal outcome trajectories under SGLT2 inhibitor therapy. The study period also partially predated the widespread adoption of angiotensin receptor–neprilysin inhibitors (ARNIs), which could have influenced outcomes.

Lifestyle and behavioral factors such as diet, physical activity, and other health-related habits were not assessed, although these variables can meaningfully influence cardiovascular prognosis. Moreover, objective measures of treatment adherence and socioeconomic status were unavailable, both of which could have contributed to heterogeneity in outcomes.

Despite these limitations, the retrospective and real-world nature of this study enhances its external validity by reflecting routine clinical practice. Future large-scale, multicenter, and prospective randomized trials are warranted to validate and extend these findings. Prospective head-to-head studies in revascularized HF populations would help determine whether subtle pharmacologic differences translate into meaningful clinical effects. Future research should also evaluate the time-varying effects of key risk factors using advanced survival modeling approaches and assess cost-effectiveness, renal outcomes, and patient-reported quality-of-life measures. Furthermore, exploring whether optimized AF management and early postoperative stabilization can modify long-term cardiovascular risk trajectories may yield valuable insights for individualized patient care.

## 7. Conclusions

In this real-world cohort of heart failure patients with a history of coronary revascularization, empagliflozin and dapagliflozin demonstrated comparable long-term cardiovascular outcomes across both preserved and reduced ejection fraction subgroups. Although no significant differences were detected, the study was not powered to exclude smaller effect sizes; thus, the results should be interpreted as reflecting an absence of observable differences rather than definitive therapeutic equivalence.

Beyond pharmacologic comparisons, advanced time-dependent modeling identified age and atrial fibrillation as the principal dynamic predictors of cardiac MACE. Both variables exhibited diminishing hazard effects over time, underscoring the importance of longitudinal risk evaluation rather than static baseline assessment.

Overall, these findings suggest that long-term cardiovascular outcomes in revascularized heart failure patients are primarily determined by individual patient characteristics—such as age, cardiac rhythm status, and comorbidity burden—rather than the specific SGLT2 inhibitor prescribed. Incorporating time-adjusted survival modeling into real-world analyses may enhance prognostic precision and support more individualized heart failure management following coronary revascularization.

## Figures and Tables

**Table 1:** Baseline characteristics of all patients treated with dapagliflozin or empagliflozin.

Variables	Dapagliflozin (*n* = 353)	Empagliflozin (*n* = 278)	*p*-Value
Age, years	62.09 ± 11.31	61.29 ± 9.65	0.388
Sex, *n* (%)			
Male	216 (61.2)	151 (54.3)	0.082
Female	137 (38.8)	127 (45.7)	0.082
BMI, kg/m^2^	27.89 ± 4.74	28.13 ± 4.71	0.687
Comorbidities, *n* (%)			
Hypertension (HT)	239 (67.7)	194 (69.8)	0.576
Diabetes mellitus (DM)	227 (64.3)	176 (63.3)	0.796
Atrial fibrillation (AF)	74 (24.3)	49 (21.5)	0.453
Smoking	61 (17.3)	47 (16.9)	0.901
Coronary artery disease (CAD), *n* (%)			
PCI	227 (64.3)	169 (60.8)	0.365
CABG	126 (35.7)	109 (39.2)	0.365
Ejection fraction (EF), %	46.0 [38.0–55.0]	49.00 [38.00–60.00]	0.052
Laboratory data			
Fasting blood glucose (FBG), mg/dL	123.20 ± 49.80	121.94 ± 41.88	0.623
HbA1c, %	6.68 ± 1.56	6.66 ± 1.35	0.616
Total cholesterol (TC), mg/dL	187.00 [150.00–222.00]	189.50 [162.00–227.00]	0.092
LDL-C, mg/dL	126.50 ± 45.07	131.49 ± 46.26	0.121
HDL-C, mg/dL	44.00 [35.75–52.00]	45.00 [38.00–57.00]	0.057
Triglycerides (TG), mg/dL	132.00 [97.00–178.00]	129.00 [102.00–181.00]	0.726
BUN (mg/dL)	20.78 ± 13.59	19.35 ± 9.19	0.952
Creatinine (mg/dL)	1.09 ± 0.77	1.00 ± 0.43	0.390
Uric acid (mg/dL)	6.35 ± 1.86	6.15 ± 1.80	0.155
NT-proBNP (pg/mL)	803.50 [330.50–1652.25]	864.00 [127.68–2502.25]	0.640
Medications, *n* (%)			
ARNi	44 (12.5)	37 (13.3)	0.753
ACEi/ARB	288 (81.6)	221 (79.5)	0.509
Beta-blocker	345 (97.7)	274 (98.6)	0.450
MRA	137 (38.8)	121 (43.5)	0.232
Diuretic	260 (73.7)	205 (73.7)	0.980
Oral anticoagulant (OAC)	82 (23.2)	75 (27)	0.280
Calcium channel blocker (CCB)	79 (22.4)	66 (23.7)	0.687
Outcomes, *n* (%)			
Hospitalization	84 (23.8)	66 (23.7)	0.987
Total mortality	68 (19.3)	55 (19.8)	0.870
Cardiac mortality	39 (11)	34 (12.2)	0.645
MACE (total mortality and Hospitalization)	91 (25.8)	73 (26.3)	0.891
Cardiac MACE	84 (23.8)	61 (21.9)	0.720
Implantable cardiac devices, *n* (%)			
ICD-CRT	29 (8.2)	23 (8.3)	0.979
Pacemaker	30 (185)	24 (8.6)	0.952
Follow-up period, months	19.67 ± 1.50	19.47 ± 1.48	0.080

Values are presented as mean ± standard deviation (SD), median [interquartile range, IQR], or number (percentage), as appropriate. *p* values indicate between-group comparisons. Abbreviations: ACEi, angiotensin-converting enzyme inhibitor; AF, atrial fibrillation; ARB, angiotensin receptor blocker; ARNi, angiotensin receptor–neprilysin inhibitor; BMI, body mass index; BUN, blood urea nitrogen; CABG, coronary artery bypass grafting; CAD, coronary artery disease; CCB, calcium channel blocker; CRT, cardiac resynchronization therapy; DM, diabetes mellitus; EF, ejection fraction; FBG, fasting blood glucose; HDL-C, high-density lipoprotein cholesterol; HT, hypertension; ICD, implantable cardioverter-defibrillator; LDL-C, low-density lipoprotein cholesterol; MACE, major adverse cardiovascular events; MRA, mineralocorticoid receptor antagonist; NT-proBNP, N-terminal pro–B-type natriuretic peptide; OAC, oral anticoagulant; PCI, percutaneous coronary intervention; SD, standard deviation; TC, total cholesterol; TG, triglyceride.

**Table 2:** Baseline characteristics of patients with EF ≥50% treated with dapagliflozin or empagliflozin.

Variables	Dapagliflozin (*n* = 153)	Empagliflozin (*n* = 152)	*p*-Value
Age, years	60.86 ± 10.59	61.89 ± 9.61	0.116
Sex, *n* (%)			
Male	89 (58.2)	75 (49.3)	0.122
Female	64 (41.8)	77 (50.7)	0.122
BMI, kg/m^2^	27.71 ± 4.16	28.48 ± 4.84	0.067
Comorbidities, *n* (%)			
Hypertension (HT)	110 (71.9)	111 (73)	0.732
Diabetes mellitus (DM)	91 (59.5)	102 (67.1)	0.167
Atrial fibrillation (AF)	26 (17)	32 (21.1)	0.366
Smoking	26 (17)	30 (19.7)	0.536
Coronary artery disease (CAD), *n* (%)			
PCI	94 (61.4)	92 (60.5)	0.870
CABG	59 (38.6)	60 (39.5)	0.870
Ejection fraction (EF), %	55.00 [52.00–60.00]	57.00 [52.00–60.00]	0.270
Laboratory data			
Fasting blood glucose (FBG), mg/dL	106.00 [96.00–132.00]	107.00 [96.25–132.00]	0.620
HbA1c, %	6.74 ± 1.67	6.74 ± 1.43	0.380
Total cholesterol (TC), mg/dL	196.00 [153.00–227.00]	196.00 [167.00–231]	0.391
LDL-C, mg/dL	130.60 ± 46.94	134.76 ± 49.59	0.315
HDL-C, mg/dL	45.00 [36.00–52.00]	46.00 [39.25–58.00]	0.132
Triglycerides (TG), mg/dL	139.50 [110.00–195.50]	132.00 [106.00–180.50]	0.513
BUN (mg/dL)	20.45 ± 15.65	19.22 ± 9.23	0.292
Creatinine (mg/dL)	1.10 ± 1.04	0.96 ± 0.35	0.920
Uric acid (mg/dL)	6.23 ± 2.01	6.34 ± 1.87	0.930
NT-proBNP (pg/mL)	745.50 [144.95–1350.50]	593.62 [86.30–2420.00]	0.314
Medications, *n* (%)			
ARNi	12 (7.8)	19 (12.5)	0.178
ACEi/ARB	131 (85.6)	117 (77)	0.053
Beta-blocker	151 (98.7)	150 (98.7)	0.995
MRA	63 (41.2)	61 (40.1)	0.853
Diuretic	122 (79.7)	119 (78.3)	0.756
Oral anticoagulant (OAC)	36 (23.5)	38 (25)	0.765
Calcium channel blocker (CCB)	29 (19)	41 (27)	0.096
Outcomes, *n* (%)			
Hospitalization	43 (28.1)	30 (19.7)	0.087
Total mortality	36 (23.5)	24 (15.8)	0.069
Cardiac mortality	19 (12.4)	12 (7.9)	0.191
MACE (total mortality and Hospitalization)	43 (28.1)	33 (21.7)	0.197
Cardiac MACE	41 (26.8)	27 (17.8)	0.140
Implantable cardiac devices, *n* (%)			
ICD-CRT	1 (0.7)	1 (0.7)	0.996
Pacemaker	8 (5.2)	7 (4.6)	0.801
Follow-up period, months	19.52 ± 1.43	19.40 ± 1.34	0.582

Values are presented as mean ± standard deviation (SD), median [interquartile range, IQR], or number (percentage), as appropriate. *p* values indicate between-group comparisons. Abbreviations: ACEi, angiotensin-converting enzyme inhibitor; AF, atrial fibrillation; ARB, angiotensin receptor blocker; ARNi, angiotensin receptor–neprilysin inhibitor; BMI, body mass index; BUN, blood urea nitrogen; CABG, coronary artery bypass grafting; CAD, coronary artery disease; CCB, calcium channel blocker; CRT, cardiac resynchronization therapy; DM, diabetes mellitus; EF, ejection fraction; FBG, fasting blood glucose; HDL-C, high-density lipoprotein cholesterol; HT, hypertension; ICD, implantable cardioverter-defibrillator; LDL-C, low-density lipoprotein cholesterol; MACE, major adverse cardiovascular events; MRA, mineralocorticoid receptor antagonist; NT-proBNP, N-terminal pro–B-type natriuretic peptide; OAC, oral anticoagulant; PCI, percutaneous coronary intervention; SD, standard deviation; TC, total cholesterol; TG, triglyceride.

**Table 3:** Baseline characteristics of patients with EF < 50% treated with dapagliflozin or empagliflozin.

Variables	Dapagliflozin (*n* = 200)	Empagliflozin (*n* = 126)	*p*-Value
Age, years	63.03 ± 11.77	62.78 ± 9.72	0.997
Sex, *n* (%)			
Male	127 (63.5)	76 (60.3)	0.564
Female	73 (36.5)	50 (39.7)	0.564
BMI, kg/m^2^	28.02 ± 5.15	27.71 ± 4.53	0.227
Comorbidities, *n* (%)			
Hypertension (HT)	129 (64.6)	83 (65.9)	0.800
Diabetes mellitus (DM)	136 (68)	74 (58.7)	0.089
Atrial fibrillation (AF)	54 (27)	30 (23.8)	0.521
Smoking	35 (17.5)	17 (13.5)	0.336
Coronary artery disease (CAD), *n* (%)			
PCI	133 (66.5)	77 (61.1)	0.322
CABG	67 (33.5)	49 (38.9)	0.322
Ejection fraction (EF), %	38.00 [35.00–45.00]	38.00 [31.75–43.00]	0.062
Laboratory data			
Fasting blood glucose (FBG), mg/dL	107.00 [95.00–129.00]	109.00 [96.50–124.00]	0.893
HbA1c, %	6.64 ± 1.48	6.55 ± 1.23	0.706
Total cholesterol (TC), mg/dL	179.50 [149.50–213.25]	188.00 [160.00–218.00]	0.279
LDL-C, mg/dL	129.54 ± 43.41	127.34 ± 41.51	0.407
HDL-C, mg/dL	44.00 [35.00–52.00]	44.00 [37.20–54.00]	0.396
Triglycerides (TG), mg/dL	120.00 [91.50–171.50]	125.00 [99.00–181.00]	0.458
BUN (mg/dL)	21.03 ± 11.75	19.51 ± 9.16	0.561
Creatinine (mg/dL)	1.08 ± 0.49	1.06 ± 0.51	0.740
Uric acid (mg/dL)	6.40 ± 1.80	6.04 ± 1.73	0.081
NT-proBNP (pg/mL)	947.20 [410.43–2013.75]	1148.80 [280.05–3008.00]	0.728
Medications, *n* (%)			
ARNi	32 (16)	18 (14.3)	0.676
ACEi/ARB	157 (78.5)	104 (82.5)	0.374
Beta-blocker	194 (97)	124 (98.4)	0.422
MRA	74 (37)	60 (47.6)	0.058
Diuretic	138 (69)	86 (68.3)	0.888
Oral anticoagulant (OAC)	46 (23)	37 (29.3)	0.199
Calcium channel blocker (CCB)	50 (25)	25 (19.9)	0.281
Outcomes, *n* (%)			
Hospitalization	41 (20.5)	36 (28.6)	0.095
Total mortality	32 (16)	31 (24.6)	0.055
Cardiac mortality	20 (10)	22 (17.5)	0.053
MACE (total mortality and Hospitalization)	48 (24)	40 (31.7)	0.125
Cardiac MACE	43 (21.5)	34 (26.9)	0.130
Implantable cardiac devices, *n* (%)			
ICD-CRT	28 (14)	22 (17.5)	0.399
Pacemaker	22 (11)	17 (13.5)	0.500
Follow-up period, months	19.79 ± 1.55	19.56 ± 1.63	0.146

Values are presented as mean ± standard deviation (SD), median [interquartile range, IQR], or number (percentage), as appropriate. *p* values indicate between-group comparisons. Abbreviations: ACEi, angiotensin-converting enzyme inhibitor; AF, atrial fibrillation; ARB, angiotensin receptor blocker; ARNi, angiotensin receptor–neprilysin inhibitor; BMI, body mass index; BUN, blood urea nitrogen; CABG, coronary artery bypass grafting; CAD, coronary artery disease; CCB, calcium channel blocker; CRT, cardiac resynchronization therapy; DM, diabetes mellitus; EF, ejection fraction; FBG, fasting blood glucose; HDL-C, high-density lipoprotein cholesterol; HT, hypertension; ICD, implantable cardioverter-defibrillator; LDL-C, low-density lipoprotein cholesterol; MACE, major adverse cardiovascular event; MRA, mineralocorticoid receptor antagonist; NT-proBNP, N-terminal pro–B-type natriuretic peptide; OAC, oral anticoagulant; PCI, percutaneous coronary intervention; SD, standard deviation; TC, total cholesterol; TG, triglyceride.

**Table 4:** Characteristics of patients with and without cardiac MACE.

Variables	Cardiac MACE (−) (*n* = 486)	Cardiac MACE (+) (*n* = 145)	*p*-Value
Age, years	61.38 ± 10.37	64.86 ± 10.98	0.004
Sex, *n* (%)			
Male	286 (58.8)	81 (55.9)	0.522
Female	200 (41.2)	64 (44.1)	0.522
BMI, kg/m^2^	28.09 ± 4.66	27.67 ± 4.94	0.413
Comorbidities, *n* (%)			
Hypertension (HT)	338 (69.5)	95 (65.5)	0.359
Diabetes mellitus (DM)	318 (65.4)	85 (58.6)	0.134
Atrial fibrillation (AF)	98 (20.2)	44 (30.3)	0.010
Smoking	81 (16.7)	27 (18.6)	0.584
Coronary artery disease (CAD), *n* (%)			
PCI	148 (62.7)	38 (55.1)	0.252
CABG	88 (37.3)	31 (44.9)	0.252
Ejection fraction (EF), %	48.00 [38.00–60.00]	45.00 [30.00–60.00]	0.037
Laboratory data			
Fasting blood glucose (FBG), mg/dL	107.00 [97.00–129.00]	106.00 [93.00–132.00]	0.921
HbA1c, %	6.66 ± 1.48	6.70 ± 1.42	0.768
Total cholesterol (TC), mg/dL	188.00 [153.00–224.50]	189.00 [164.50–225.50]	0.178
LDL-C, mg/dL	128.55 ± 46.47	129.17 ± 42.86	0.775
HDL-C, mg/dL	45.00 [36.00–54.00]	44.00 [37.00–58.00]	0.887
Triglycerides (TG), mg/dL	129.00 [99.00–178.00]	132.00 [98.50–175.50]	0.639
BUN (mg/dL)	19.36 ± 10.96	22.76 ± 14.01	0.001
Creatinine (mg/dL)	0.99 ± 0.41	1.24 ± 1.09	0.006
Uric acid (mg/dL)	6.31 ± 1.82	6.49 ± 1.93	0.051
NT-proBNP (pg/mL)	674.40 [150.80–1571.00]	1289.00 [617.12–5443.00]	0.001
Medications, *n* (%)			
ARNi	57 (11.7)	24 (16.6)	0.128
ACEi/ARB	393 (80.9)	116 (80)	0.817
Beta-blocker	475 (97.7)	144 (99.3)	0.223
MRA	191 (39.3)	67 (46.2)	0.138
Diuretic	352 (72.4)	113 (77.9)	0.187
Oral anticoagulant (OAC)	105 (21.6)	52 (35.9)	0.001
Calcium channel blocker (CCB)	119 (24.5)	26 (17.9)	0.100
Implantable cardiac devices, *n* (%)			
ICD-CRT	35 (7.5)	17 (10.4)	0.250
Pacemaker	38 (8.1)	16 (9.8)	0.534
SGLT2i, *n* (%)			
Dapagliflozin	269 (55.3)	84 (57.9)	0.583
Empagliflozin	217 (44.7)	61 (42.1)	0.583

Values are presented as mean ± standard deviation (SD), median [interquartile range, IQR], or number (percentage), as appropriate. *p* values indicate comparisons between patients with and without cardiac MACE. Abbreviations: ACEi, angiotensin-converting enzyme inhibitor; AF, atrial fibrillation; ARB, angiotensin receptor blocker; ARNi, angiotensin receptor–neprilysin inhibitor; BMI, body mass index; BUN, blood urea nitrogen; CABG, coronary artery bypass grafting; CAD, coronary artery disease; CCB, calcium channel blocker; CRT, cardiac resynchronization therapy; DM, diabetes mellitus; EF, ejection fraction; FBG, fasting blood glucose; HDL-C, high-density lipoprotein cholesterol; HT, hypertension; ICD, implantable cardioverter-defibrillator; LDL-C, low-density lipoprotein cholesterol; MACE, major adverse cardiovascular event; MRA, mineralocorticoid receptor antagonist; NT-proBNP, N-terminal pro–B-type natriuretic peptide; OAC, oral anticoagulant; PCI, percutaneous coronary intervention; SD, standard deviation; TC, total cholesterol; TG, triglyceride.

**Table 5:** Univariable Cox regression analysis for prediction of cardiac MACE.

Variables	*p*-Value	HR	95% CI
Age	0.001	1.026	1.011–1.042
Atrial fibrillation (AF)	0.040	1.452	1.018–2.207
Oral anticoagulant use (OAC)	0.001	2.029	1.357–3.034
Ejection fraction (EF)	0.068	0.987	0.973–1.001
NT-proBNP	0.007	1.001	1.001–1.002
Serum creatinine	0.041	1.141	1.005–1.294
SGLT2i type (Dapagliflozin vs. Empagliflozin)	0.968	1.007	0.725–1.398

Abbreviations: AF, atrial fibrillation; CI, confidence interval; EF, ejection fraction; HR, hazard ratio; NT-proBNP, N-terminal pro–B-type natriuretic peptide; OAC, oral anticoagulant; SGLT2i, sodium–glucose cotransporter-2 inhibitor.

**Table 6:** Results of the multivariable Cox regression model corrected for proportional hazards violation (AF-stratified).

Variables	Coefficient (B)	Standard Error (SE)	Wald	*p*-Value	HR (Exp[B])	95% CI (Exp[B])
Age	0.737	0.098	56.127	<0.001	2.089	1.723–2.533
Serum creatinine	0.157	0.139	1.289	0.256	1.170	0.892–1.536
NT-proBNP	0.000	0.000	0.585	0.444	1.000	1.000–1.000
TIMEAGE (Age × Time interaction)	−0.038	0.005	54.121	<0.001	0.963	0.953–0.973

Abbreviations: AF, atrial fibrillation; CI, confidence interval; HR, hazard ratio; NT-proBNP, N-terminal pro–B-type natriuretic peptide; SE, standard error.

## Data Availability

The data presented in this study are available on reasonable request from the corresponding author. The data are not publicly available due to privacy and ethical restrictions.
